# Casein sIgE avidity and milk basophil reactivity in children with cow's milk allergy

**DOI:** 10.1111/pai.70464

**Published:** 2026-08-18

**Authors:** Otso Nieminen, Kati Palosuo, Terhi Savinko, Victoria Hinkkanen, Piia Karisola, Mika J. Mäkelä

**Affiliations:** ^1^ Department of Allergology Helsinki University Hospital Helsinki Finland; ^2^ University of Helsinki Helsinki Finland

**Keywords:** avidity, basophil activation test, casein, children, cow's milk allergy, heated milk, IgE

## Abstract

**Background:**

In our previous study, casein specific IgE (sIgE) was identified as the best predictor for heated milk (HM) tolerance. However, reaction severity and cumulative eliciting dose in oral food challenges (OFC) remain difficult to predict. The use of milk basophil activation test (BAT) and sIgE avidity has not been previously studied for this purpose in HM allergy without the presence of wheat matrix. Consequently, we aimed to investigate whether they could improve prediction of fresh milk (FM) and HM OFC outcomes, reaction severity, and cumulative eliciting dose.

**Methods:**

1–18‐year‐old Finnish children with suspected IgE‐mediated cow's milk allergy underwent OFCs with both FM (*N* = 160) and HM (*N* = 138). sIgE to milk and its allergens was measured. Milk BAT using whole blood and casein sIgE avidity measurements using a thiocyanate‐based ELISA assay were performed.

**Results:**

Milk BAT showed better performance in discriminating allergy and tolerance in FM OFCs (*N* = 57, area under the curve (AUC) 0.946) than in HM OFCs (*N* = 42, AUC 0.686). Casein sIgE avidity did not differ between HM allergic and tolerant children (*N* = 56). Neither assay was associated with reaction severity or cumulative eliciting dose in FM or HM OFCs.

**Conclusion:**

Milk BAT performed well in FM allergy prediction, while in HM OFCs its diagnostic accuracy was modest and did not exceed that of casein sIgE. Casein sIgE avidity did not improve prediction of FM or HM OFC outcome, reaction severity, or cumulative dose. The avidity assay was also inherently limited by the requirement for casein sIgE levels of >1kU/L.

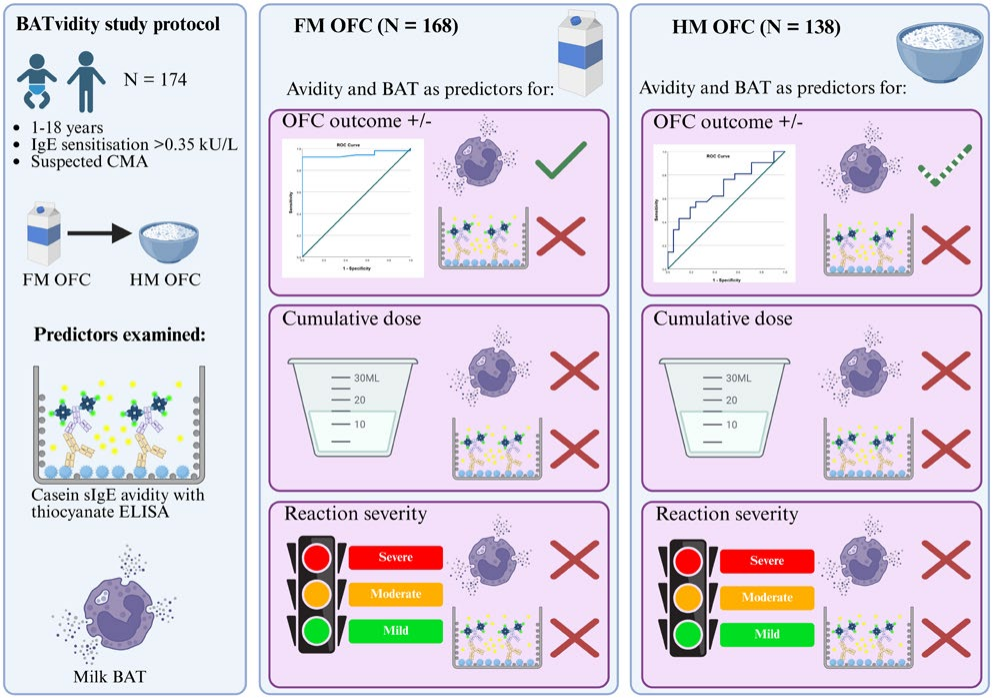

## Graphical abstract

Milk BAT accurately predicts fresh milk allergy but is more limited in heated milk allergy. Casein sIgE avidity does not differ between heated milk allergic and tolerant patients. Casein sIgE avidity and milk BAT do not predict reaction severity or cumulative dose in positive fresh or heated milk oral food challenges. Created in BioRender. Nieminen, O. L. T. (2026) https://BioRender.com/huvj8bm.

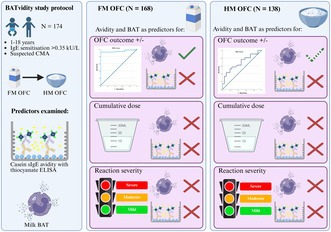

**Key message**

Milk BAT appears to be an accurate predictor of FM allergy while its accuracy in HM allergy diagnostics is more modest, and it cannot predict reaction severity or cumulative eliciting dose in either FM or HM OFCs. Casein sIgE avidity did not demonstrate additional diagnostic value in this cohort using the current assay methodology. While milk BAT can help in FM allergy diagnostics, casein BAT might prove more useful in HM allergy diagnostics.

## 1 INTRODUCTION

The need for predicting the cumulative eliciting dose and the reaction severity in positive oral food challenges (OFC) has long been acknowledged. However, traditional tests, such as specific IgE (sIgE) and skin prick tests (SPT), have proven inadequate for this purpose.^1^, ^2^ The advent of functional assays, such as basophil activation test (BAT), together with the more experimental methods for epitope and antibody avidity determination, has provided some promising results in this regard.

In peanut and baked egg allergies, BAT has been used to recognize allergy from tolerance^3^, ^4^ and to identify the risk for severe allergic reactions and low cumulative doses.^5^, ^6^ However, data on BAT and baked milk (BM) allergy has long remained scarce.^7^, ^8^ Most previous cow's milk allergy (CMA) studies have explored either how BAT predicts fresh milk (FM) or BM OFC outcome^9^, ^10^, ^11^, ^12^, ^13^ or how it predicts FM allergy outgrowth.^14^, ^15^, ^16^ Just recently, three studies exploring the use of BAT in BM allergy^17^, ^18^, ^19^ emerged attesting to the interest on the subject but no study has evaluated BAT in relation to heated milk (HM) lacking wheat matrix. Furthermore, validation of BM results in different populations is still required.^20^

We hypothesized that IgE avidity could serve as a marker of clinical reactivity, as higher‐avidity IgE is thought to reflect more mature, affinity‐selected immune responses that may promote more efficient FcƐRI cross‐linking and stronger effector cell activation, thereby translating into more severe or lower‐threshold allergic reactions. Affinity refers to the binding strength between an individual antibody's antigen binding site and its epitope, whereas avidity is the cumulative strength of antibody–antigen interaction.^21^ High‐affinity antibodies may form more stable allergen‐IgE complexes that can activate mast cells and basophils at lower allergen concentrations^22^ which could explain why patients with similar sIgE concentrations experience different reactions. Avidity's correlation to reaction severity in peanut allergy^23^ and its capability to differentiate heated egg allergic from tolerant children^24^ have been published. However, studies that would have quantified antibody avidity to predict FM, BM, or HM OFC outcome, reaction severity, or cumulative eliciting dose are lacking.

In our previous study,^25^ we investigated HM allergy in IgE‐mediated CMA and could not connect sIgE or SPT with reaction severity or cumulative eliciting dose. Thus, we now further investigated the possibilities for predicting them using milk BAT and casein sIgE avidity. Casein sIgE avidity was chosen as casein is the heat‐stable allergen in cow's milk,^26^ and has been found as the best predictor for BM tolerance.^18^, ^25^, ^27^ To our knowledge, avidity has not previously been tested quantitatively in HM allergy, and only one recent study evaluating BAT in BM OFCs has examined the connection between BAT and reaction severity and cumulative eliciting dose^19^ yet no corresponding data on HM allergy exists.

## 2 METHODS

### 2.1 Primary and secondary outcomes

The primary outcomes were to explore the usefulness of milk BAT to predict HM OFC outcome, reaction severity, and cumulative eliciting dose; to establish cutoff values for milk BAT to predict HM OFC outcome; and to evaluate the ability of casein sIgE avidity to predict HM OFC outcome, reaction severity, and cumulative eliciting dose. Secondary outcomes were to use milk BAT and casein sIgE avidity to predict FM OFC outcome, reaction severity, and cumulative eliciting dose. However, this study should be considered primarily exploratory, as no formal power calculations were performed because of the relatively small expected sample size.

### 2.2 Study population

The inclusion and exclusion criteria, the study protocol, and the OFC protocols have been described in our previous study in detail.^25^ Briefly, 160 Finnish children (58.8% male) aged 1–18 years underwent an FM OFC to confirm their CMA status, and 138 (60.1% male) children with positive FM OFCs were then challenged with HM (rice porridge typical for Finnish food culture, heated in oven at 175°C for 90 min, clinical allergenicity validated in our previous study^25^). Challenge outcome defined by PRACTALL,^28^ reaction severity defined by modified Sampson's severity score (mSSS),^29^ and cumulative eliciting dose (mg) were recorded, SPTs to FM (skim milk, Arla, Sipoo, Finland) and HM (milk boiled for 15 min) performed, and sIgE to milk (f2), casein (Bos d 8), alpha‐lactalbumin (Bos d 4), beta‐lactoglobulin (Bos d 5), and BSA (bovine serum albumin, Bos d 6) measured using ImmunoCAP (Thermo Fisher, Uppsala, Sweden). The serum samples used for sIgE determination were frozen to be used later in casein sIgE avidity measurements, and BAT was performed whenever possible. Blood for BAT was usually drawn on the same visit with sIgE measurements.

### 2.3 Ethical Statement

Ethical approval for the study was given by the Helsinki University Hospital of Children and Adolescents Ethics Committee. A signed written informed consent from a guardian and each child ≥6 years of age was required for participation. The study adhered to the Declaration of Helsinki.

### 2.4 Basophil activation tests

Venous blood samples for BAT were collected into K2EDTA tubes (Greiner Bio‐One, Austria), stored at +22°C, and analyzed within 24 h.^20^, ^30^ Flow CAST kit and milk allergen (both from BUHLMANN Laboratories, Schönenbuch, Switzerland) were used to investigate basophil activation. Briefly, whole blood was incubated with milk allergen (BAG‐F2, 22.5 ng/mL) for 25 min at 37°C in the presence of stimulation buffer containing interleukin‐3 and fluorescently labeled antibodies against CCR3 and CD63. Red blood cells were lysed and cells suspended in wash buffer for flow cytometry (FACSLyric, BD, New Jersey). Basophils were gated SSClowCCR3+ (median of 933 cells gated, interquartile range (IQR): 594–981 cells) and CD63 was used as an activation marker. Non‐responders (activation rate <10% with the positive control FcƐRI monoclonal antibody) and patients with spontaneous activation of basophils (baseline activation >5%) were excluded from the study.

### 2.5 Casein sIgE avidity measurement protocol with thiocyanate‐based ELISA assay

**FIGURE 1:**
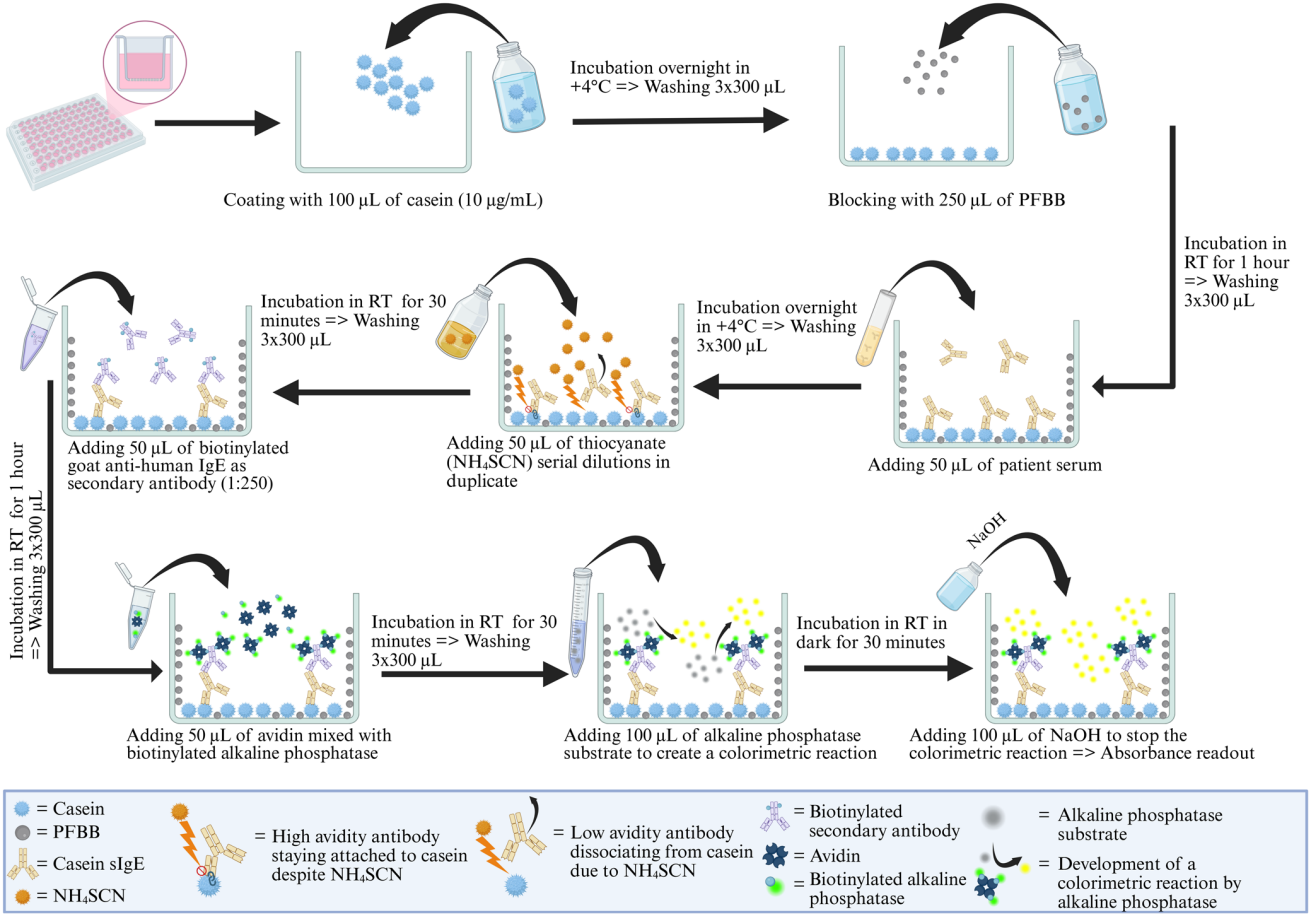
The thiocyanate‐based ELISA assay protocol for casein‐sIgE avidity measurement. The protocol can be found in detail under the methods section. NaOH, sodium hydroxide; NH_4_SCN, Thiocyanate; PFBB, Protein‐free blocking buffer; RT, Room temperature. Created in BioRender. Nieminen, O. L. T. (2026) https://BioRender.com/4dajocg.

We estimated the cumulative binding strength of casein sIgE using a thiocyanate‐based ELISA assay. Thiocyanate is a chaotropic agent that disrupts antibody–antigen binding and thus allows for indirect determination of avidity^31^ by measuring absorbance. The protocol was modified from a previously published method for Ara h 2 avidity measurement in peanut allergy,^23^ and is presented in Figure 1. Patients with casein sIgE ≥1kU/L were chosen for avidity measurements as suggested for peanut,^32^, ^33^ as low casein sIgE resulted in absorbance values not reliably separable from background. To be considered reliable and included in analysis, baseline absorbance values (0 M thiocyanate) had to be ≥0.2 to twice exceed the background absorbance (mean 0.080, range 0.060–0.091) across all the avidity measurements.

96‐well ELISA plates (Nunc‐Immuno Plate Maxi‐Sorp™) were coated with 100 μL of purified casein (Bos d 8, compliments of Thermo Fisher) diluted to 10 μg/mL in 0.05 M carbonate buffer per well. The next day the wells were washed (PBS, Biotop + Tween 0.05%), blocked with 250 μL of blocking buffer (Pierce™ Protein‐Free Blocking Buffer, Thermo Scientific + Tween 0.05%), and washed. Then, 50 μL of patient serum diluted in blocking buffer to 1–5 kU/L was added to each well. After washing, 50 μL of serial thiocyanate (NH_4_SCN) dilutions (0, 1, 2, 3, 4 M; diluted in blocking buffer) were added in duplicate to the wells as per the pipetting template (Data S1). After washing, 50 μL of biotinylated goat anti‐human IgE antibody (BA‐3040, 1:250 dilution in blocking buffer, Vector Laboratories Inc., Newark, CA, USA) was added as secondary antibody. After washing, 50 μL of biotinylated alkaline phosphatase and avidin (Vectastain ABC‐Ap Kit, Vector Laboratories, Inc.) diluted in PBS as per the manufacturer's instructions was added to each well. Avidin's four binding sites for biotin and their strong binding^34^ contributed to choosing avidin‐biotin system to amplify the colorimetric reaction. After a final wash, 100 μL of alkaline phosphatase substrate (2 PnPP tablets in 10 mL of 1× diethanolamine substrate, Thermo Scientific) was added to each well. The reaction was stopped with 100 μL of 1 M NaOH. The results were read with Multiskan FC (Thermo Scientific) at a wavelength of 405 nm.

### 2.6 Avidity determination

**FIGURE 2:**
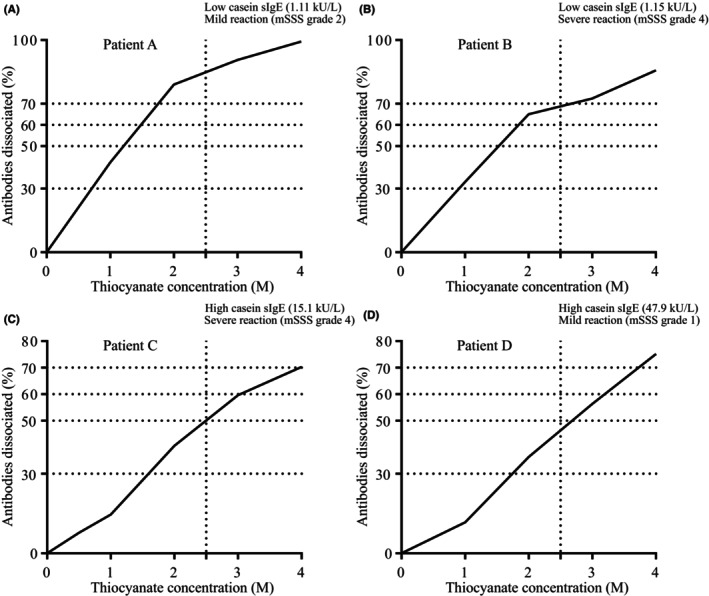
Casein sIgE avidity graphs. These graphs demonstrate that avidity is similar between patients regardless of casein sIgE levels or HM reaction severity. (A) low casein sIgE, mild reaction, (B) low casein sIgE, severe reaction, (C) high casein sIgE, severe reaction, (D) high casein sIgE, mild reaction. HM, Heated milk; mSSS, Modified Sampson's severity score.^29^

Percentual decrease of absorbance values compared to baseline (0 M thiocyanate) was plotted against the thiocyanate molarity (Figure 2). The thiocyanate molarity at which absorbance was reduced by 30%, 50%, 60%, and 70% from baseline (representative of similar percentage of antibodies being progressively dissociated from casein) was determined from the plot and used as a quantifiable surrogate for antibody avidity.^31^ Also, the reduction of absorbance at the fixed thiocyanate concentration of 2.5 M (reflecting percentage of dissociated antibodies at this concentration) was determined to estimate overall antibody stability.

### 2.7 Statistical analysis, figures, and tables

SPSS Inc. (Chicago, IL) versions 28 and 31 software were used for statistical analyses. Figures were made with SPSS, Microsoft Power point (version 2509), Inkscape software (version 1.2.2), GraphPad Prism (GraphPad Software, version 6.07 Boston, Massachusetts USA), and Biorender (www.BioRender.com). Tables were prepared using Microsoft Word (version 2509).

The normality of data was tested with Shapiro–Wilk test due to sample size <50 in each group. Statistical difference between groups was tested with Mann–Whitney *U* test (two groups) and Kruskal‐Wallis test (three or more groups) if the data was nonnormally distributed. The significance of *p*‐values was further confirmed with Hodges‐Lehman estimate. For normally distributed data, an independent samples *T*‐test was used, and the two‐sided significance values were used after testing for equal variances. A *p*‐value of <0.05 was considered significant. If statistically significant differences were noted for avidity measurements, Bonferroni corrections were applied by multiplying the *p*‐value with the number of tests performed to correct for multiple testing. Receiver operating characteristics (ROC) analysis was used to determine cutoff values. Optimal cutoff values were chosen based on Youden's index.

## 3 RESULTS

### 3.1 Study population and reaction severity

**FIGURE 3:**
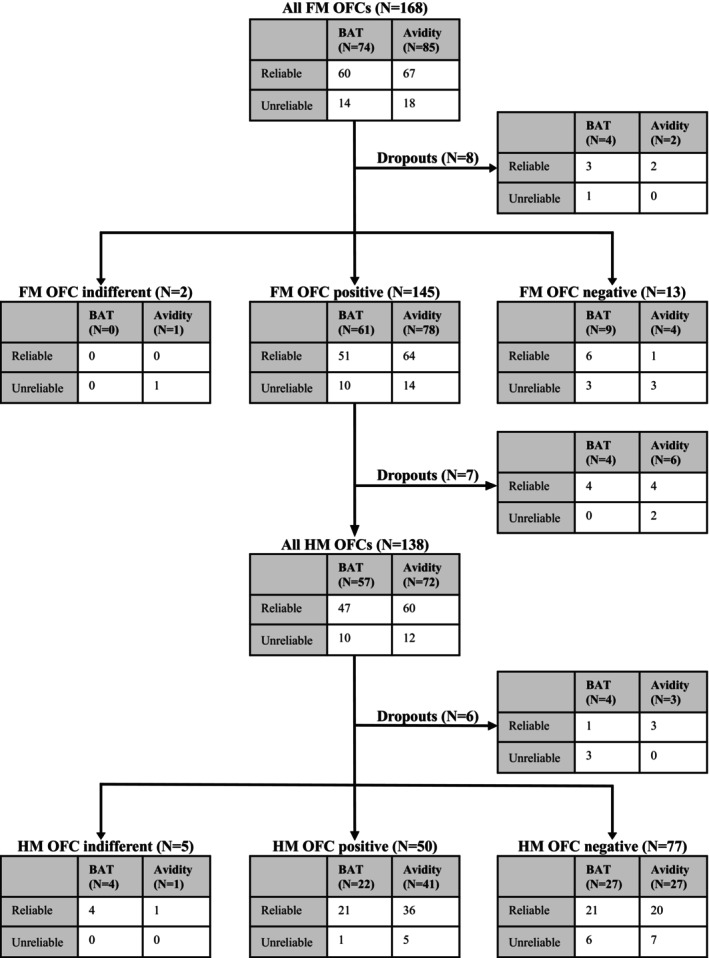
The flowchart of the study. The number of patients (i.e. OFCs) accompanied by the number of performed milk BAT and casein sIgE avidity measurements at each step of the study is shown. The number of reliable and unreliable basophil activation test and antibody avidity measurements are further presented. For avidity, unreliable was defined as baseline absorbance <0.2 and for basophils as non‐responding (activation rate of <10% with the positive control FcƐRI monoclonal antibody) or hyperreactive (activation rate >5% without stimulation) test results. BAT, basophil activation test; FM, Fresh milk; OFC, Oral food challenge; HM, Heated milk.

The details concerning the study population in whole have previously been reported in detail.^25^ The subgroup demographics according to OFC status in the groups with reliable BAT and avidity measurements appear in eTable S1A–D. Briefly, 160 FM OFCs were performed, of which 145 (86%) were positive. Of the subsequent 138 HM OFCs, 50 (36%) were positive. In positive FM OFCs, 79% of reactions were mild, 10% moderate, and 11% severe when evaluated with mSSS.^29^ In positive HM OFCs, the corresponding percentages were 66%, 18%, and 16%. The reactions elicited and their severities in the subgroups are presented in eTable S2. Figure 3 summarizes the distribution of avidity and BAT measurements according to OFC status. For BAT, 74 measurements were performed, of which nine (12.2%) were considered non‐responders (FcƐRI antibody‐induced activation: 2.0%–7.8%) and five (6.8%) spontaneously activated (background activation: 33.4%–73.4%). In total, avidity measurements were performed for 85 patients, of which 67 (78.8%) were considered reliable. Casein sIgE was significantly higher in children with reliable avidity measurements (median 8.52kU/L, IQR 3.78–14.7kU/L) compared to children with unreliable avidity measurements (median 1.46kU/L, IQR 1.21–2.48kU/L) (*p* < .001) (eTable S4B).

eTable S3A demonstrates that no selection bias was introduced because of limited application of BAT in the population. eTable S3B shows that successful avidity measurements were associated with higher sIgE levels and more clinically relevant CMA and other allergic conditions compared to children without successful avidity measurements. This was to be expected as avidity was not measured for children with casein sIgE concentration <1kU/L. eTable S4 demonstrates similar tendency when comparing children with reliable BAT and avidity measurements to children who were excluded from BAT and avidity analysis because of unreliable results.

### 3.2 Milk BAT, casein sIgE avidity, and FM OFC outcomes

**FIGURE 4:**
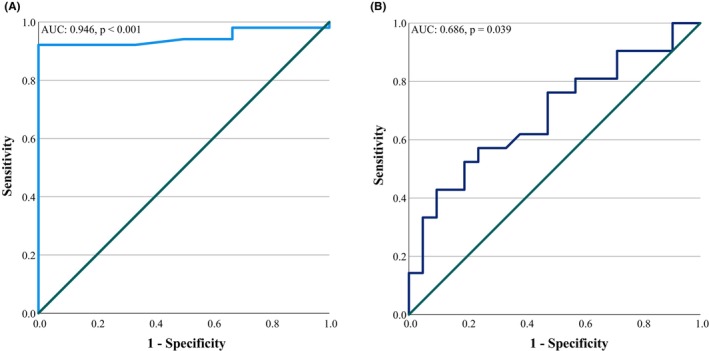
The receiver operating characteristic curves for basophil activation tests for fresh milk (A) and heated milk (B). The cutoff values, areas under the curves (AUC), and other characteristics are shown in Table 2.

For milk BAT, the median reactivity was significantly greater in positive FM OFCs (median 34.3%) compared to negative FM OFCs (median 2.3%) (Table 1A). In ROC analysis, BAT had an area under the curve (AUC) of 0.946. The cutoff of 3.4% had 100% specificity and 92% sensitivity, while the cutoff of 2.0% produced 33% specificity and 96% sensitivity (Table 2A, Figure 4A) Among children with positive FM OFCs (*N* = 51), milk BAT reactivity was not correlated with cumulative dose or reaction severity (mSSS).^29^ Furthermore, BAT reactivity did not differ across the three (mild, moderate, severe) mSSS^29^ severity categories (*p* = .351), between mild and moderate‐to‐severe FM reactions (*p* = .180), or cumulative dose categories (*p* = .318).

**TABLE 1:** Milk BAT reactivity and avidity results in FM OFCs (A) and HM OFCs (B).

(A)	FM OFC+	FM OFC−	*p*	HL estimate (95% CI)	Effect size
**BAT reactivity (%)**	*N* = 51	*N* = 6			
Median (IQR)	34.3 (12.2 to 63.6)	2.3 (1.7 to 2.7)	<0.001^a^	−32.1 (−61.1 to −10.6)	0.47
Mean (95% CI)	NA	2.2 (1.7 to 2.8)	NA	NA	NA
Non‐responders, *N* (%)	6 (9.8)	3 (33.3)	0.084	NA	NA
Hyperreactive, *N* (%)	4 (6.6)	0 (0.0)	1.00	NA	NA
**Avidity**					
Baseline absorbance	*N* = 64	*N* = 1			
Median (IQR)	0.35 (0.22 to 0.46)	0.49 (0.49 to 0.49)	NA	NA	NA
NH_4_SCN at 30% (M)	*N* = 64	*N* = 1			
Median (IQR)	1.49 (1.25 to 1.90)	1.43 (1.43 to 1.43)	NA	NA	NA
NH_4_SCN at 50% (M)	*N* = 60	*N* = 1			
Median (IQR)	2.33 (1.85 to 2.87)	2.07 (2.07 to 2.07)	NA	NA	NA
NH_4_SCN at 60% (M)	*N* = 52	*N* = 1			
Median (IQR)	2.64 (2.18 to 3.18)	2.69 (2.69 to 2.69)	NA	NA	NA
Mean (95% CI)	2.71 (2.54 to 2.88)	NA	NA	NA	NA
NH_4_SCN at 70% (M)	*N* = 46	*N* = 1			
Median (IQR)	3.13 (2.83 to 3.70)	3.66 (3.66 to 3.66)	NA	NA	NA
Dissociation at 2.5 M (%)	*N* = 64	*N* = 1			
Median (IQR)	51.7 (41.7 to 62.7)	56.8 (56.8 to 56.8)	NA	NA	NA
Mean (95% CI)	51.6 (48.0 to 55.2)	NA	NA	NA	NA

(B)	HM OFC+	HM OFC−	*p*	HL estimate (95% CI)	Effect size
**BAT reactivity (%)**	*N* = 21	*N* = 21			
Median (IQR)	42.3 (21.2 to 75.0)	23.1 (5.8 to 39.3)	0.039^a^	−18.6 (−37.6 to −0.4)	0.32
Mean (95% CI)	45.7 (32.0 to 59.3)	27.0 (17.0 to 36.9)	0.026^a^	NA	−0.713
Non‐responders, *N* (%)	0 (0.0)	3 (11.1)	0.242	NA	NA
Hyperreactive, *N* (%)	1 (4.5)	3 (11.1)	0.617	NA	NA
**Avidity**					
Baseline absorbance	*N* = 36	*N* = 20			
Median (IQR)	0.37 (0.23 to 0.47)	0.27 (0.20 to 0.40)	0.137	−0.053 (−0.139 to 0.023)	NA
NH_4_SCN at 30% (M)	*N* = 36	*N* = 20			
Median (IQR)	1.48 (1.28 to 1.84)	1.55 (1.32 to 2.09)	0.351	0.12 (−0.14 to 0.45)	NA
Mean (95% CI)	1.55 (1.37 to 1.72)	1.73 (1.44 to 2.02)	0.257	NA	NA
NH_4_SCN at 50% (M)	*N* = 35	*N* = 17			
Median (IQR)	2.38 (1.89 to 2.85)	2.07 (1.88 to 2.95)	0.838	−0.07 (−0.45 to 0.37)	NA
Mean (95% CI)	2.39 (2.16 to 2.63)	2.36 (2.04 to 2.68)	0.855	NA	NA
NH_4_SCN at 60% (M)	*N* = 31	*N* = 14			
Median (IQR)	2.74 (2.19 to 3.22)	2.54 (2.16 to 3.19)	0.787	−0.06 (−0.50 to 0.38)	NA
Mean (95% CI)	2.73 (2.49 to 2.97)	2.70 (2.37 to 3.04)	0.891	NA	NA
NH_4_SCN at 70% (M)	*N* = 26	*N* = 13			
Median (IQR)	3.15 (2.83 to 3.70)	3.13 (2.71 to 3.68)	0.826	0.035 (−0.36 to 0.43)	NA
Mean (95% CI)	NA	3.19 (2.89 to 3.49)	NA	NA	NA
Dissociation at 2.5 M (%)	*N* = 36	*N* = 20			
Median (IQR)	51.6 (42.7 to 61.5)	50.8 (38.4 to 62.2)	0.483	−2.0 (−11.4 to 6.8)	NA
Mean (95% CI)	52.6 (47.8 to 57.4)	49.3 (42.1 to 56.6)	0.424	NA	NA

*Note*: Mean values are only shown for groups that are normally distributed. If the data is non‐normally distributed, only median is shown. Significance of *p*‐values in non‐parametric tests is further confirmed with Hodges‐Lehmann test. Cohen's d was used to estimate effect size for the *T*‐test. For Mann Whitney *U* test, effect size was calculated as *r* = Z/$\sqrt{N}$. No statistical testing was meaningful for avidity parameters in FM OFCs due to only 1 child in the FM OFC negative group. Milk BAT non‐responders were defined as <10% activation rate with the positive control FcƐRI monoclonal antibody while hyperreactivity was defined as >5% baseline activation without stimulation. NH_4_SCN at 30%, 50%, 60%, and 70% refer to the calculated thiocyanate concentration at which baseline absorbance was reduced by the corresponding percentage. This percentage was interpreted as the amount of dissociated casein sIgE, and the thiocyanate concentrations were used as quantifiable surrogates for avidity. Dissociation at 2.5 M records the percentage of dissociated antibodies from casein at thiocyanate concentration 2.5 M.

Abbreviations: BAT, basophil activation test; CI, confidence interval; FM, fresh milk; HL, Hodges‐Lehmann; HM, heated milk; IQR, interquartile range; M, molarity; NA, not applicable; NH_4_SCN, thiocyanate; OFC, oral food challenge; r, effect size; Z, the test statistic from Mann Whitney *U* test.

^a^ Statistically significant (*p* < .05) difference (without Bonferroni correction).

**TABLE 2:** The capability of milk BAT reactivity to predict FM OFC (A, *N* = 57) and HM OFC (B, *N* = 42) outcome.

	AUC (95% CI)	*p*	Cutoff (%)	Specificity % (95% CI)	Sensitivity % (95% CI)	PPV (%)	NPV (%)	LR+	LR−
**(A)**									
BAT reactivity									
100%SP	0.946 (0.888–1.00)	<0.001^a^	3.4	100.0 (78.8–100^b^)	92.2 (71.9–100^b^)	60.0	100.0	NA	0.08
95% SE	0.946 (0.888–1.00)	<0.001^a^	2.0	33.3 (21.1–45.5)	96.1 (75.4–100^b^)	92.5	50.0	1.4	0.1
Optimal cutoff	0.946 (0.888–1.00)	<0.001^a^	3.4	100.0 (78.8–100^b^)	92.2 (71.9–100^b^)	60.0	100.0	NA	0.08
**(B)**									
BAT reactivity									
95% SP	0.686 (0.524–0.848)	0.039^a^	67.6	95.2 (75.6–100^b^)	33.3 (21.7–44.9)	87.5	58.8	7.0	0.7
95% SE	0.686 (0.524–0.848)	0.039^a^	2.2	9.5 (3.3–15.7)	95.2 (75.6–100^b^)	51.3	66.7	1.1	0.5
Optimal cutoff^c^	0.686 (0.524–0.848)	0.039^a^	39.3/41.2/52.4	76.2 (58.7–93.7)/81.0 (62.9–99.1)/90.5 (71.4–100^b^)	57.1 (41.9–72.3)/52.4 (37.9–66.9)/42.9 (29.8–56.0)	70.6/73.3/81.8	64.0/63.0/61.3	2.4/2.8/4.5	0.6/0.6/0.6

*Note*: Cutoffs for 95% specificity, 95% sensitivity, and optimal performance as per Youden's index are shown. 95% confidence intervals for specificity and sensitivity have been calculated as $X\pm 1.96\times \sqrt{\frac{X-\left(1-X\right)}{n_{0/1}}}$, where X = specificity/sensitivity, n_0_ = number of non‐allergic subjects (for specificity calculation), and n_1_ = number of allergic subjects (for sensitivity calculation).^35^

Abbreviations: AUC, area under the curve; BAT, basophil activation test; CI, confidence interval; LR+, positive likelihood ratio; LR−, negative likelihood ratio; NPV, negative predictive value; PPV, positive predictive value.

^a^ Statistically significant difference (*p* < .05).

^b^ The upper bound of the 95% CI exceeds 100% and has been truncated to 100%.

^c^ Three cutoffs had equal Youden's indexes (0.33) and are thus all shown.

Table 1A summarizes all the studied avidity parameters. In positive FM OFCs, reaction severity (mSSS)^29^ or cumulative eliciting dose was not correlated with any of these avidity parameters. Also, there was no difference in any avidity parameter when compared between the mSSS^29^ severity categories (mild, moderate, severe and mild vs. moderate‐to‐severe) or cumulative dose categories.

### 3.3 Milk BAT, casein sIgE avidity, and HM OFC outcomes

There were 21 and 21 BAT results in positive and negative HM OFCs, respectively. BAT reactivity was significantly greater in positive (median 42.3%) compared to negative HM OFCs (median 23.1%) (Table 1B). In ROC analysis, BAT reactivity had an AUC of 0.686. Cutoff value of 67.6% had 95% specificity and 33% sensitivity, while the cutoff value 2.2% had 10% specificity and 95% sensitivity (Table 2B, Figure 4B). Regarding positive HM OFCs, BAT was not correlated with cumulative eliciting dose or reaction severity (mSSS)^29^ nor was there any difference in BAT reactivity between different reaction severity groups (mild, moderate, severe: *p* = .757 and mild vs. moderate‐to‐severe: *p* = .913).

The measured avidity parameters in HM OFC positive (*N* = 36) and HM OFC negative (*N* = 20) groups are summarized in Table 1B. No significant differences were seen in any of the avidity parameters between the groups. Only antibody dissociation rate of 70% correlated with cumulative eliciting dose (*p* = .023, r = −0.445). However, the scatter plot eFigure (S1) was not convincing (wide 95% confidence interval, small *R* ^2^ value, only one patient with a large cumulative dose) and statistical significance was lost after Bonferroni correction. Furthermore, there was no difference in any of the avidity parameters between the cumulative dose categories. No avidity parameter correlated with reaction severity and there was no significant difference in avidity parameters between the three mSSS^29^ reaction severity categories or mild and moderate‐to‐severe reactions. Figure 2 demonstrates that the avidity curves are similar regardless of casein sIgE level and HM OFC reaction severity.

## 4 DISCUSSION

This follow‐up study evaluated the diagnostic utility of milk BAT and casein sIgE avidity in challenge‐proven CMA and, to our knowledge, for the first time also in relation to HM allergy. Milk BAT reactivity could accurately differentiate FM tolerant from allergic children but did not surpass casein sIgE in predicting HM OFC outcome.^25^ Furthermore, BAT did not correlate with cumulative eliciting dose or reaction severity in either FM or HM OFC. We also found that casein sIgE avidity did not differ between HM allergic and tolerant children and did not correlate with cumulative dose or reaction severity in positive FM or HM OFCs.

Our finding that milk BAT reactivity accurately predicts FM OFC outcome (AUC 0.946) concurs with previous studies. However, the cutoff value of 3.4% (100% specificity, 92% sensitivity) appears lower than the previously proposed cutoffs of 9%–10% (100% specificity, 67%–100% sensitivity) in CMA.^9^, ^10^ Compared to other food allergies, a slightly higher cutoff was proposed in peanut allergy, whereas a similar cutoff was reported in egg allergy.^36^ In respect to our previous study with a different population,^37^ BAT seems superior to casein and milk sIgE in FM allergy prediction.

The role of milk BAT in HM allergy diagnostics, however, appeared somewhat more limited. We found that milk BAT (AUC 0.686) did not surpass casein sIgE (AUC 0.786) in discriminating HM tolerance from allergy in the same population.^25^ Recently, Bartha et al. described both milk and baked milk BAT as the most accurate predictors for BM allergy.^17^ However, only 39% of children in their BM tolerant group had OFC‐confirmed CMA^17^ emphasizing their different approach compared to our study on exclusively OFC‐confirmed CMA. Concurring with our results, Ford et al. reported an AUC of 0.69 for milk BAT reactivity and an AUC of 0.78 for casein sIgE.^12^ Additionally, Dominguez et al. recently described casein sIgE as the better biomarker (AUC 0.83) compared to milk BAT (AUC 0.74) to predict BM allergy when also including cost‐effectiveness in the analysis.^18^

We could not observe an association between milk BAT reactivity and cumulative eliciting dose in either FM or HM allergy. This could be because we did not examine BAT sensitivity which has been linked to threshold dose in egg allergy, while BAT reactivity appears to be more connected to reaction severity.^6^ However, we could not connect BAT reactivity to FM or HM reaction severity either, which attests to the complex interplay of various factors determining the outcome of an allergen exposure. Boyd et al. recently reported that milk BAT discriminated severe and non‐severe reactions in BM OFCs, while cumulative dose could only be predicted in FM OFCs.^19^ For FM OFCs, Røisgård et al. published that casein BAT sensitivity correlated with both reaction severity and cumulative dose.^2^ Furthermore, in peanut and egg allergies, BAT has been reported to identify children at risk of severe allergic reactions at low cumulative doses,^5^, ^6^ but not all studies have been able to show a connection between reaction severity and BAT.^38^ Thus, milk BAT does not appear to be very effective in HM allergy diagnostics and the high costs, resource requirements, and the 24‐h time limit associated with it pose further challenges for its application.^18^, ^30^

Higher antibody affinity has been hypothesized to lead to smaller threshold doses,^39^ but we could not show any reliable connection between casein sIgE avidity and cumulative dose or reaction severity in either FM or HM OFCs nor was there connection to HM OFC outcome. Interestingly, children in HM OFC positive and negative groups with reliable avidity measurements had similar sIgE levels to milk and all its allergens (eTable S1D). This is likely to be a consequence of filtering out the children with low sIgE concentrations in the HM OFC negative group as their casein sIgE level was not high enough for reliable avidity determination (eTable S3B). As exactly these children whose allergy status cannot be reliably evaluated due to low‐to‐intermediate sIgE concentration would have benefitted the most from new diagnostic methods it seems that casein sIgE avidity has little diagnostic value. However, these results can currently be generalized only to children whose casein sIgE concentration is >1kU/L. The application of avidity measurements is further complicated by the increased likelihood for measurement failure with low casein sIgE concentrations (eTable S4B). Only one study on CMA has focused on affinity's capability to differentiate between degrees of milk tolerance^40^ and no study has quantified avidity or explored its connection to reaction severity or cumulative dose. Two other studies have explored antibody binding intensity in CMA but not avidity or affinity specifically.^41^, ^42^ However, studies on peanut have found higher avidity in allergic compared to sensitized patients,^33^ and weak correlation between Ara h 2 sIgE avidity and reaction severity.^23^ In heated egg allergy, ovomucoid sIgE avidity was higher in allergic compared to tolerant patients, and anaphylactic reactions were characterized by higher sIgE‐to‐avidity ratio compared to non‐anaphylactic reactions.^24^ Nevertheless, other IgE characteristics have proven more useful compared to avidity in peanut allergy diagnostics.^43^ Thus, the significance of avidity alone appears meager.

The avidity measurements' lack of usefulness could be due to many factors, such as the indirect method of using colorimetric reaction to measure avidity, the lack of lateral diffusion in an ELISA assay hampering IgE crosslinking compared to basophils in vivo,^21^ and the inability to account for the role of other factors affecting the development of an allergic reaction. These include epitope diversity; number of linear epitopes; sIgE concentration; presence of cellular inhibitory effects; BAT reactivity and sensitivity measuring cellular activation and IgE cross‐linking; and allergen concentration.^6^, ^8^, ^11^, ^33^, ^40^, ^41^, ^42^, ^43^ Consequently, examining avidity alone in vitro simplifies the complex cascade of events leading to an allergic reaction and does not accurately reflect the situation in vivo.

As a strength, this is the first study to quantitatively examine the role of casein sIgE avidity in FM and HM allergy and the first study to investigate the capability of milk BAT to predict reaction severity and cumulative eliciting dose in HM allergy. However, we acknowledge that there are certain limitations to our study. First, due to COVID‐19 and laboratory shut down, the number of BATs performed especially in the negative FM OFCs was lower than expected. Second, we did not analyze casein BAT which, compared to milk BAT, could have provided more relevant information in HM allergy as casein is the heat‐stable milk allergen^26^ and the results with casein BAT have been promising in FM allergy.^2^ Third, not every patient had a serum sample available for avidity measurements and samples with casein sIgE concentration <1.0kU/L could not be reliably measured with this method. Thus, the significance of avidity in children with low casein sIgE concentrations cannot be evaluated based on these results. Fourth, the highest antibody dissociation rates of 60%–70% were not reached in every child probably indicating high avidity of these antibodies and an inadequate highest thiocyanate concentration. Coupled with the relatively small sample size that did not allow for reliable multivariate analysis, the results of this study should be regarded as exploratory and no definitive conclusions may be drawn, but it seems that antibody avidity does not add clinically relevant information to decision making in CMA.

In summary, milk BAT accurately identified FM allergic children while its utility in HM allergy did not surpass that of casein sIgE in this cohort. Furthermore, milk BAT was not associated with reaction severity or cumulative eliciting dose in either FM or HM OFCs. Casein sIgE avidity did not differentiate HM allergic from tolerant patients and was not associated with cumulative eliciting dose or reaction severity in HM of FM OFCs.

## AUTHOR CONTRIBUTIONS

**Otso Nieminen:** Conceptualization; methodology; data curation; investigation; validation; formal analysis; funding acquisition; visualization; writing – original draft; writing – review and editing. **Piia Karisola:** Methodology; resources; writing – review and editing. **Victoria Hinkkanen:** Methodology; writing – review and editing; investigation. **Mika J. Mäkelä:** Conceptualization; methodology; supervision; funding acquisition; project administration; resources; writing – review and editing. **Terhi Savinko:** Writing – review and editing; writing – original draft; investigation; resources. **Kati Palosuo:** Conceptualization; methodology; supervision; project administration; resources; writing – review and editing.

## FUNDING INFORMATION

This study was funded by the University of Helsinki, the Helsinki University Hospital Research funds, Sigrid Jusélius Foundation, the Finnish Medical Foundation, the Pediatric Research Foundation, and the Allergy Research Foundation.

## CONFLICT OF INTEREST STATEMENT

The casein used to coat the ELISA plates was provided by Thermo Fisher free of charge. The authors have no other conflicts of interest pertaining to this article.

## Supporting information


**eFigure S1.** The scatter plot for correlation between cumulative eliciting dose in positive heated milk oral food challenges and the 70% avidity point demonstrates there is no reliable connection between avidity and cumulative eliciting dose (wide 95% confidence interval, small R^2^ values, only one patient with high cumulative dose).

**Data S1.** Pipetting template.

**eTable S1.** Baseline characteristics, median values of SPTs and sIgEs, and age‐related cumulative doses according to FM OFC status for children with reliable BAT (A) and avidity measurements (B) and according to HM OFC status for children with reliable BAT (C) and avidity measurements (D). A BAT result (tested at milk allergen concentration 22.5 ng/mL) was considered unreliable if activation rate of basophils was <10% with the positive control FcƐRI monoclonal antibody (non‐responder) or if spontaneous activation at baseline without stimulation exceeded 5% (hyperreactive). Avidity measurements were considered unreliable if the baseline absorbance was <0.2. Other food allergies screened for: egg, any nut, fish, wheat, soy.

**eTable S2.** The severity of reactions and the rate of different symptoms experienced during positive FM OFCs (A) and positive HM OFCs (B) in children with reliable BAT results and reliable avidity measurements. Severity assessment is according to the modified Sampson's severity score, and anaphylaxis is defined according to Sampson et al.

**eTable S3.** Baseline characteristics, median values of SPTs and sIgEs, age‐related HM cumulative doses, and OFC outcomes according to BAT status (A) and avidity status (B). BAT status was determined either as successful or not successful (including non‐responsive, hyperreactive, or not performed). A BAT result was considered non‐responsive if activation rate of basophils was <10% with the positive control FcƐRI monoclonal antibody and hyperreactive if spontaneous activation at baseline without stimulation exceeded 5%. Avidity measurements were considered successful or not successful (unreliable or not performed). Unreliable avidity results had baseline absorbances <0.2.

**eTable S4.** Baseline sIgE, age at HM OFC, and both FM and HM OFC outcomes in the groups with (A) successful and excluded, nonreliable BAT measurements (*N* = 70) and (B) successful and excluded, nonreliable avidity measurements (*N* = 82).

## Data Availability

Research data are not shared.
