# Supplementary material for: Casein sIgE avidity and milk basophil reactivity in children with cow's milk allergy
Source: Pediatr Allergy Immunol. 2026 Aug 18;37(8):e70464. doi: 10.1111/pai.70464 (PMC13486920; doi:10.1111/pai.70464)
Supplement: Supplementary file 1 [file PAI-37-e70464-s003.pdf]

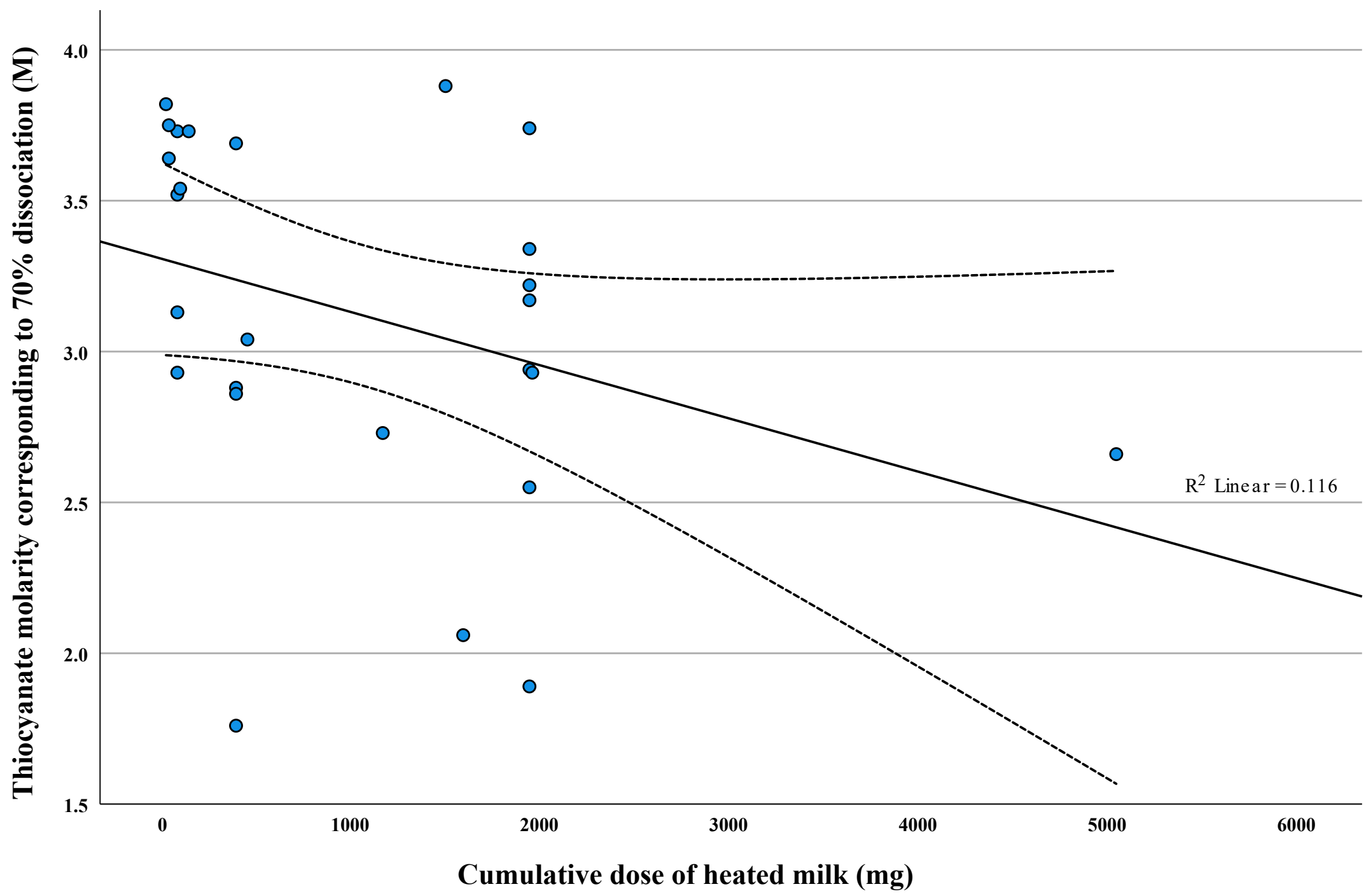

eFigure S1. The scatter plot for correlation between cumulative eliciting dose in positive heated milk oral food challenges and the 70% avidity point demonstrates there is no reliable connection between avidity and cumulative eliciting dose (wide 95% confidence interval, small R^2 values, only one patient with high cumulative dose).
