# Supplementary material for: Casein sIgE avidity and milk basophil reactivity in children with cow's milk allergy
Source: Pediatr Allergy Immunol. 2026 Aug 18;37(8):e70464. doi: 10.1111/pai.70464 (PMC13486920; doi:10.1111/pai.70464)
Supplement: Supplementary file 2 [file PAI-37-e70464-s001.docx]

**A)**

|  | | | FM OFC– (N = 6) | FM OFC+ (N = 51) | p-values |
| --- | --- | --- | --- | --- | --- |
| Median sIgE, kU/L (IQR) | | |  |  |  |
|  | | Milk (f2) | 1.28 (0.52–2.53) | 4.89 (1.82–12.5) | **0.005*** |
|  | | Casein (Bos d 8) | 0.84 (0.26–1.64) | 2.13 (0.67–6.15) | 0.089 |
|  | | Alpha-lactalbumin (Bos d 4) | 0.39 (0.33–0.66) | 1.55 (0.27–4.23) | 0.111 |
|  | | Beta-lactoglobulin (Bos d 5) | 0.55 (0.03–1.12) | 1.15 (0.25–6.38) | 0.105 |
|  | | BSA (Bos d 6) | 0.09 (0.02–0.26) | 0.01 (0.0–0.02) | **0.018*** |
| Median SPT wheal size, mm (IQR) | | |  |  |  |
|  | | Fresh milk | 4.5 (3.0–8.0) | 9.0 (7.0–11.3) | **0.011*** |
|  | | Heated milk | 4.0 (2.3–7.0) | 5.5 (5.0–8.0) | 0.093 |
|  | | Egg | 2.0 (0.0–5.3) | 4.0 (0.0–5.0) | 0.632 |
|  | | Wheat | 0.0 (0.0–0.8) | 0.0 (0.0–0.0) | 0.786 |
|  | | Gliadin | 0.0 (0.0–0.0) | 0.0 (0.0–0.0) | 0.826 |
|  | | Peanut | 3.5 (0.0–5.5) | 4.0 (0.0–8.3) | 0.578 |
|  | | Cashew nut | 4.5 (0.0–8.5) | 4.0 (0.0–10.0) | 0.908 |
| Baseline characteristics | | |  |  |  |
| Sex, male, N (%) | | | 3 (50.0) | 27 (52.9) | 1.00 |
| Median age (years) at HM OFC (IQR) | | | 1.9 (1.2–3.1) | 3.4 (–1.4–7.4) | 0.168 |
| Atopic dermatitis, N (%)^†^ | | | 0 (0.0) / 4 (66.7) / 2 (33.3) | 3 (5.9) / 18 (35.3) / 30 (58.8) | 0.310 |
| Asthma, N (%)^†^ | | | 5 (83.3) / 0 (0.0) / 1 (16.7) | 35 (68.6) / 6 (11.8) / 10 (19.6) | 0.639 |
| Pollen allergy, N (%)^†^ | | | 5 (83.3) / 0 (0.0) / 1 (16.7) | 26 (51.0) / 1 (2.0) / 24 (47.1) | 0.320 |
| Animal dust allergy, N (%)^†^ | | | 5 (83.3) / 0 (0.0) / 1 (16.7) | 31 (60.8) / 1 (2.0) / 19 (37.3) | 0.549 |
| Other food allergies, median (IQR) | | | 1.5 (0.8–2.3) | 1.0 (0.0–2.0) | 0.694 |
|  | Atopic dermatitis in family, N (%) | | 1 (16.7) | 35 (68.6) | **0.022*** |
|  | Asthma in family, N (%) | | 1 (16.7) | 17 (34.0) | 0.652 |
|  | Allergies in family, N (%) | | 4 (66.7) | 41 (82.0) | 0.586 |
|  | Tobacco smoking in family, N (%) | | 0 (0.0) | 0 (0.0) | NA |
| Median HM OFC cumulative dose, mg | | |  |  |  |
| ≤ 3-year-old (IQR) | | | 5035 (3485–5035) | 75 (15–618) | NA |
| >3-year-old (IQR) | | | 5035 (5035–5035) | 385 (90–1160) | NA |

**B)**

|  | | | FM OFC– (N = 1) | FM OFC+ (N = 64) | p-values |
| --- | --- | --- | --- | --- | --- |
| Median sIgE, kU/L (IQR) | | |  |  |  |
|  | | Milk (f2) | 12.3 | 14.7 (8.44–26.5) | NA |
|  | | Casein (Bos d 8) | 2.2 | 8.54 (3.86–14.7) | NA |
|  | | Alpha-lactalbumin (Bos d 4) | 14.9 | 3.79 (0.86–13.2) | NA |
|  | | Beta-lactoglobulin (Bos d 5) | 0.57 | 3.99 (1.01–7.37) | NA |
|  | | BSA (Bos d 6) | 0.49 | 0.02 (0.0–0.19) | NA |
| Median SPT wheal size, mm (IQR) | | |  |  | NA |
|  | | Fresh milk | 6.0 | 9.0 (7.0–12.0) | NA |
|  | | Heated milk | 7.0 | 7.0 (5.0–9.0) | NA |
|  | | Egg | 0.0 | 4.5 (3.0–6.0) | NA |
|  | | Wheat | 0.0 | 0.0 (0.0–3.3) | NA |
|  | | Gliadin | 0.0 | 0.0 (0.0–0.0) | NA |
|  | | Peanut | 8.0 | 5.0 (0.0–10.3) | NA |
|  | | Cashew nut | 9.0 | 7.0 (0.0–10.0) | NA |
| Baseline characteristics | | |  |  |  |
| Sex, male, N (%) | | | 1 (100) | 41 (64.1) | NA |
| Median age (years) at HM OFC (IQR) | | | 6.0 | 3.2 (1.8–5.9) | NA |
| Atopic dermatitis, N (%)^†^ | | | – / – / 1 (100) | 3 (4.7) / 16 (25.0) / 45 (70.3) | NA |
| Asthma, N (%)^†^ | | | – / – / 1 (100) | 42 (65.6) / 4 (6.3) / 18 (28.1) | NA |
| Pollen allergy, N (%)^†^ | | | – / – / 1 (100) | 31 (48.4) / 2 (3.1) / 31 (48.4) | NA |
| Animal dust allergy, N (%)^†^ | | | – / 1 (100) / – | 38 (59.4) / 3 (4.7) / 23 (35.9) | NA |
| Other food allergies, median (IQR) | | | 1.0 | 2.0 (1.0–2.0) | NA |
|  | Atopic dermatitis in family, N (%) | | 0 (0.0) | 44 (68.8) | NA |
|  | Asthma in family, N (%) | | 0 (0.0) | 19 (29.7) | NA |
|  | Allergies in family, N (%) | | 0 (0.0) | 49 (77.8) | NA |
|  | Tobacco smoking in family, N (%) | | 0 (0.0) | 3 (4.7) | NA |
| Median HM OFC cumulative dose, mg | | |  |  |  |
| ≤ 3-year-old (IQR) | | | – | 230 (15–695) | NA |
| >3-year-old (IQR) | | | 3485 | 385 (75–695) | NA |

**C)**

|  | | | HM OFC– (N = 21) | HM OFC+ (N = 21) | p-values |
| --- | --- | --- | --- | --- | --- |
| Median sIgE, kU/L (IQR) | | |  |  |  |
|  | | Milk (f2) | 2.78 (1.22–8.61) | 8.20 (3.10–23.0) | **0.006*** |
|  | | Casein (Bos d 8) | 1.23 (0.39–3.40) | 4.02 (1.36–9.15) | **0.016*** |
|  | | Alpha-lactalbumin (Bos d 4) | 1.23 (0.13–3.53) | 3.39 (0.39–14.5) | 0.078 |
|  | | Beta-lactoglobulin (Bos d 5) | 0.44 (0.22–1.26) | 2.85 (0.83–7.34) | **0.011** |
|  | | BSA (Bos d 6) | 0.0 (0.0–0.02) | 0.01 (0.0–0.03) | 0.620 |
| Median SPT wheal size, mm (IQR) | | |  |  |  |
|  | | Fresh milk | 8.0 (6.0–10.0) | 9.0 (7.0–12.0) | 0.226 |
|  | | Heated milk | 5.0 (4.0–6.0) | 7.0 (5.0–8.5) | **0.004*** |
|  | | Egg | 4.0 (0.0–5.0) | 3.0 (0.0–6.0) | 0.630 |
|  | | Wheat | 0.0 (0.0–0.0) | 0.0 (0.0–3.0) | 0.555 |
|  | | Gliadin | 0.0 (0.0–0.0) | 0.0 (0.0–0.0) | 0.452 |
|  | | Peanut | 3.0 (0.0–5.0) | 7.0 (0.0–12.0) | **0.014*** |
|  | | Cashew nut | 7.0 (0.0–10.0) | 5.0 (0.0–12.0) | 0.810 |
| Baseline characteristics | | |  |  |  |
| Sex, male, N (%) | | | 10 (47.6) | 12 (57.1) | 0.758 |
| Median age (years) at HM OFC (IQR) | | | 2.55 (1.41–5.37) | 3.59 (1.89–7.43) | 0.285 |
| Atopic dermatitis, N (%)^†^ | | | 1 (4.8) / 8 (38.1) / 12 (57.1) | 1 (4.8) / 6 (28.6) / 14 (66.7) | 0.803 |
| Asthma, N (%)^†^ | | | 17 (81.0) / 3 (14.3) / 1 (4.8) | 13 (61.9) / 3 (14.3) / 5 (23.8) | 0.202 |
| Pollen allergy, N (%)^†^ | | | 12 (57.1) / – / 9 (42.9) | 10 (47.6) / 1 (4.8) / 10 (47.6) | 0.539 |
| Animal dust allergy, N (%)^†^ | | | 16 (76.2) / 1 (4.8) / 4 (19.0) | 10 (47.6) / – / 11 (52.4) | 0.059 |
| Other food allergies, median (IQR) | | | 2.0 (0.0–2.0) | 2.0 (0.5–2.0) | 0.769 |
|  | Atopic dermatitis in family, N (%) | | 15 (71.4) | 14 (66.7) | 1.00 |
|  | Asthma in family, N (%) | | 7 (33.3) | 7 (33.3) | 1.00 |
|  | Allergies in family, N (%) | | 17 (81.0) | 17 (81.0) | 1.00 |
|  | Tobacco smoking in family, N (%) | | 0 (0.0) | 0 (0.0) | NA |
| Median HM OFC cumulative dose, mg | | |  |  |  |
| ≤ 3-year-old (IQR) | | | 1935 (1935–3485) | 385 (230–1943) | NA |
| >3-year-old (IQR) | | | 5035 (3485–5035) | 1908 (385–1935) | NA |

**D)**

|  | | | HM OFC– (N = 20) | HM OFC+ (N = 36) | p-values |
| --- | --- | --- | --- | --- | --- |
| Median sIgE, kU/L (IQR) | | |  |  |  |
|  | | Milk (f2) | 12.9 (9.72–30.7) | 13.5 (8.15–23.8) | 0.614 |
|  | | Casein (Bos d 8) | 6.59 (3.24–13.7) | 8.25 (3.65–13.7) | 0.878 |
|  | | Alpha-lactalbumin (Bos d 4) | 4.25 (0.86–7.56) | 4.00 (1.79–15.3) | 0.432 |
|  | | Beta-lactoglobulin (Bos d 5) | 3.99 (1.10–8.02) | 3.58 (0.80–7.27) | 0.573 |
|  | | BSA (Bos d 6) | 0.04 (0.01–1.40) | 0.02 (0.00–0.14) | 0.172 |
| Median SPT wheal size, mm (IQR) | | |  |  |  |
|  | | Fresh milk | 8.0 (7.0–9.0) | 9.0 (6.0–12.0) | 0.466 |
|  | | Heated milk | 5.0 (5.0–7.5) | 8.0 (5.0–9.0) | **0.04*** |
|  | | Egg | 4.5 (3.0–6.3) | 5.0 (0.8–6.0) | 0.662 |
|  | | Wheat | 0.0 (0.0–4.0) | 0.0 (0.0–4.5) | 0.941 |
|  | | Gliadin | 0.0 (0.0–0.0) | 0.0 (0.0–0.0) | 0.973 |
|  | | Peanut | 4.0 (0.0–7.5) | 5.5 (3.0–12.0) | 0.126 |
|  | | Cashew nut | 8.0 (0.0–10.3) | 7.0 (1.0–10.0) | 0.860 |
| Baseline characteristics | | |  |  |  |
| Sex, male, N (%) | | | 12 (60.0) | 25 (69.4) | 0.561 |
| Median age (years) at HM OFC (IQR) | | | 3.4 (1.4–5.2) | 3.4 (2.2–7.8) | 0.573 |
| Atopic dermatitis, N (%)^†^ | | | 1 (5.0) / 5 (25.0) / 14 (70.0) | 1 (2.8) / 9 (25.0) / 26 (72.2) | 0.911 |
| Asthma, N (%)^†^ | | | 15 (75.0) / 2 (10.0) / 3 (15.0) | 23 (63.9) / 2 (5.6) / 11 (30.6) | 0.400 |
| Pollen allergy, N (%)^†^ | | | 10 (50.0) / 2 (10.0) / 8 (40.0) | 17 (47.2) / – / 19 (52.8) | 0.132 |
| Animal dust allergy, N (%)^†^ | | | 11 (55.0) / 2 (10.0) / 7 (35.0) | 22 (61.1) / – / 14 (38.9) | 0.155 |
| Other food allergies, median (IQR) | | | 2.0 (1.0–2.8) | 2.0 (1.0–2.0) | 0.662 |
|  | Atopic dermatitis in family, N (%) | | 12 (60.0%) | 26 (72.2) | 0.383 |
|  | Asthma in family, N (%) | | 5 (25.0) | 13 (36.1) | 0.552 |
|  | Allergies in family, N (%) | | 14 (70.0) | 30 (83.3) | 0.313 |
|  | Tobacco smoking in family, N (%) | | 1 (5.0) | 2 (5.6) | 1.00 |
| Median HM OFC cumulative dose, mg | | |  |  |  |
| ≤ 3-year-old (IQR) | | | 1935 (1935–1935) | 1373 (385–1935) | NA |
| >3-year-old (IQR) | | | 5035 (2279–5035) | 385 (101–1935) | NA |

eTable S1. Baseline characteristics, median values of SPTs and sIgEs, and age-related cumulative doses according to FM OFC status for children with reliable BAT (A) and avidity measurements (B) and according to HM OFC status for children with reliable BAT (C) and avidity measurements (D). A BAT result (tested at milk allergen concentration 22.5ng/mL) was considered unreliable if activation rate of basophils was <10% with the positive control FcƐRI monoclonal antibody (non-responder) or if spontaneous activation at baseline without stimulation exceeded 5% (hyperreactive). Avidity measurements were considered unreliable if the baseline absorbance was <0.2. Other food allergies screened for: egg, any nut, fish, wheat, soy.

Missing information: A) SPT from 1 patient in the positive group, asthma in family for 1 patient in the positive group, B) SPT from 2 patients in the positive group, C) SPT from 2 patients in the negative group, asthma in family for 1 patient in the negative group, D) SPT from 2 patients in the negative group.

sIgE: specific immunoglobulin E, kU/L: kilounits per liter, IQR: interquartile range; BSA, bovine serum albumin, mm: millimeter, HM OFC: heated milk oral food challenge, HM OFC+: positive HM OFC, HM OFC–: negative HM OFC, NA: not applicable.

* Denotes a statistically significant (p < 0.05) difference (without Bonferroni correction).

^†^ Never/previously/currently
