# Supplementary material for: Casein sIgE avidity and milk basophil reactivity in children with cow's milk allergy
Source: Pediatr Allergy Immunol. 2026 Aug 18;37(8):e70464. doi: 10.1111/pai.70464 (PMC13486920; doi:10.1111/pai.70464)
Supplement: Supplementary file 3 [file PAI-37-e70464-s004.docx]

|  | | BAT successful (N = 57) | BAT excluded (nonresponder or hyperreactive, N=13) | p-values |
| --- | --- | --- | --- | --- |
| Median sIgE, kU/L (IQR) | |  |  |  |
|  | Milk (f2) | 3.43 (1.57–12.2) | 10.5 (0.97–20.5) | 0.419 |
|  | Casein (Bos d 8) | 1.64 (0.64–5.73) | 1.61 (0.18–0.933) | 0.946 |
|  | Alpha-lactalbumin (Bos d 4) | 1.23 (0.30–4.15) | 2.44 (0.05–8.97) | 0.868 |
|  | Beta-lactoglobulin (Bos d 5) | 1.06 (0.25–3.92) | 4.56 (0.19–6.99) | 0.361 |
|  | BSA (Bos d 6) | 0.01 (0.00–0.03) | 0.85 (0.00–4.58) | **0.005*** |
| Median age (years) at HM OFC (IQR) | | 3.4 (1.6–6.2) | 1.9 (1.4–2.6) | 0.062 |
| FM OFC positive, N (%) | | 51 (89.5) | 10 (76.9) | 0.353 |
| HM OFC positive, N (%) | | 21 (50.0) | 1 (14.3) | 0.112 |

**A)**

For median age at HM OFC, N=47 for successful BAT and N=10 for excluded BAT.

For HM OFC positive, N=42 for successful BAT and N=7 for excluded BAT.

**B)**

|  | | Avidity successful (N = 65) | Avidity excluded (nonreliable, N=17) | p-values |
| --- | --- | --- | --- | --- |
| Median sIgE, kU/L (IQR) | |  |  |  |
|  | Milk (f2) | 14.5 (8.47–25.8) | 2.95 (1.43–9.11) | **<0.001*** |
|  | Casein (Bos d 8) | 8.52 (3.78–14.7) | 1.46 (1.21–2.48) | **<0.001*** |
|  | Alpha-lactalbumin (Bos d 4) | 3.81 (0.98–13.7) | 0.59 (0.21–5.64) | **0.027*** |
|  | Beta-lactoglobulin (Bos d 5) | 3.93 (0.86–7.34) | 0.81 (0.16–4.61) | **0.029*** |
|  | BSA (Bos d 6) | 0.02 (0.00–0.29) | 0.01 (0.00–0.18) | 0.229 |
| Median age (years) at HM OFC (IQR) | | 3.4 (2.1–5.4) | 2.1 (1.6–3.1) | 0.082 |
| FM OFC positive, N (%) | | 61 (98.5) | 14 (82.4) | **0.027*** |
| HM OFC positive, N (%) | | 36 (64.3) | 5 (41.7) | 0.130 |

For median age at HM OFC, N=60 for successful avidity and N=12 for excluded avidity.

For HM OFC positive, N=56 for successful avidity and N=12 for excluded avidity.

eTable S4. Baseline sIgE, age at HM OFC, and both FM and HM OFC outcomes in the groups with (A) successful and excluded, nonreliable BAT measurements (N=70) and (B) successful and excluded, nonreliable avidity measurements (N=82).

sIgE: specific IgE, BAT: basophil activation test, BSA: bovine serum albumin, HM: heated milk, OFC: oral food challenge, FM: fresh milk, *: statistically significant (p<0.05) difference (without Bonferroni correction).
