# Supplementary material for: Casein sIgE avidity and milk basophil reactivity in children with cow's milk allergy
Source: Pediatr Allergy Immunol. 2026 Aug 18;37(8):e70464. doi: 10.1111/pai.70464 (PMC13486920; doi:10.1111/pai.70464)
Supplement: Supplementary file 4 [file PAI-37-e70464-s005.docx]

**A)**

|  |  | | | BAT successful (N = 57) | No BAT or BAT unreliable (N = 103) | p-values |
| --- | --- | --- | --- | --- | --- | --- |
|  | Median sIgE, kU/L (IQR) | | |  |  |  |
|  | | | Milk (f2) | 3.43 (1.57–12.2) | 6.00 (1.87–14.8) | 0.334 |
|  | | | Casein (Bos d 8) | 1.64 (0.64–5.73) | 1.25 (0.30–8.52) | 0.558 |
|  | | | Alpha-lactalbumin (Bos d 4) | 1.23 (0.30–4.08) | 2.37 (0.35–6.00) | 0.384 |
|  | | | Beta-lactoglobulin (Bos d 5) | 1.06 (0.25–3.92) | 1.10 (0.22–4.99) | 0.763 |
|  | | | BSA (Bos d 6) | 0.01 (0.00–0.03) | 0.02 (0.00–0.38) | **0.01*** |
|  | Median SPT wheal size, mm (IQR) | | |  |  |  |
|  | | | Fresh milk | 8.5 (6.0–11.0) | 7.5 (5.0–11.0) | 0.248 |
|  | | | Heated milk | 5.0 (5.0–8.0) | 5.0 (4.0–8.0) | 0.630 |
|  | | | Egg | 4.0 (0.0–5.0) | 4.0 (0.0–5.0) | 0.799 |
|  | | | Wheat | 0.0 (0.0–0.0) | 0.0 (0.0–3.0) | 0.461 |
|  | | | Gliadin | 0.0 (0.0–0.0) | 0.0 (0.0–0.0) | 0.331 |
|  | | | Peanut | 4.0 (0.0–7.8) | 3.0 (0.0–7.0) | 0.343 |
|  | | | Cashew nut | 4.0 (0.0–10.0) | 5.0 (0.0–8.0) | 0.882 |
|  | Baseline characteristics | | |  |  |  |
|  | Sex, male, N (%) | | | 30 (52.6) | 64 (62.1) | 0.247 |
|  | Median age (years) at HM OFC (IQR) | | | 3.4 (1.6–6.2) | 2.6 (1.7–5.2) | 0.430 |
|  | Atopic dermatitis, N (%)^†^ | | | 3 (5.3) / 22 (38.6) / 32 (56.1) | 12 (11.7) / 37 (35.9) / 54 (52.4) | 0.457 |
|  | Asthma, N (%)^†^ | | | 40 (70.2) / 6 (10.5) / 11 (19.3) | 83 (80.6) / 2 (1.9) / 18 (17.5) | 0.061 |
|  | Pollen allergy, N (%)^†^ | | | 31 (54.4) 1 (1.8) / 25 (43.9) | 60 (58.3) / 3 (2.9) / 40 (38.8) | 0.852 |
|  | Animal dust allergy, N (%)^†^ | | | 36 (63.2) 1 (1.8) / 20 (35.1) | 72 (69.9) / 6 (5.8) / 25 (24.3) | 0.228 |
|  | Other food allergies, median (IQR) | | | 1.0 (0.0–2.0) | 1.0 (0.0–2.0) | 0.494 |
|  | | Atopic dermatitis in family, N (%) | | 36 (63.2) | 68 (66.0) | 0.732 |
|  | | Asthma in family, N (%) | | 18 (32.1) | 31 (30.1) | 0.858 |
|  | | Allergies in family, N (%) | | 45 (78.9) | 77 (74.8) | 0.556 |
|  | | Tobacco smoking in family, N (%) | | 0 (0.0) | 12 (11.7) | **0.005*** |
|  | Median HM OFC cumulative dose, mg | | |  |  |  |
|  | ≤ 3-year-old (IQR) | | | 1935 (482–1946) | 1935 (1935–1980) | 0.246 |
|  | >3-year-old (IQR) | | | 2655 (1506–5035) | 3485 (415–5035) | 0.739 |
| FM OFC positive, N (%) | | | | 51 (89.5) | 94 (93.1) | 0.548 |
| HM OFC positive, N (%) | | | | 21 (43.8) | 29 (31.5) | 0.122 |

**B)**

|  |  | | | Avidity successful (N = 65) | No successful avidity (N = 95) | p-values |
| --- | --- | --- | --- | --- | --- | --- |
|  | Median sIgE, kU/L (IQR) | | |  |  |  |
|  | | | Milk (f2) | 14.5 (8.47-25.8) | 2.20 (1.10-4.54) | **<0.001*** |
|  | | | Casein (Bos d 8) | 8.52 (3.78-14.65) | 0.57 (0.19-1.23) | **<0.001*** |
|  | | | Alpha-lactalbumin (Bos d 4) | 3.81 (0.98-13.7) | 1.03 (0.17-3.20) | **<0.001*** |
|  | | | Beta-lactoglobulin (Bos d 5) | 3.93 (0.86-7.34) | 0.52 (0.16-1.87) | **<0.001*** |
|  | | | BSA (Bos d 6) | 0.02 (0.00-0.29) | 0.01 (0.00-0.08) | **0.034*** |
|  | Median SPT wheal size, mm (IQR) | | |  |  |  |
|  | | | Fresh milk | 9.0 (7.0-12.0) | 7.0 (5.0-9.0) | **0.010*** |
|  | | | Heated milk | 7.0 (5.0-9.0) | 5.0 (4.0-7.0) | **<0.001*** |
|  | | | Egg | 4.0 (3.0-6.0) | 3.0 (0.0-5.0) | **0.004*** |
|  | | | Wheat | 0.0 (0.0-3.0) | 0.0 (0.0-0.0) | **0.007*** |
|  | | | Gliadin | 0.0 (0.0-0.0) | 0.0 (0.0-0.0) | 0.247 |
|  | | | Peanut | 5.0 (0.0-10.0) | 0.0 (0.0-6.0) | **0.004*** |
|  | | | Cashew nut | 7.0 (0.0-10.0) | 3.0 (0.0-8.0) | **0.003*** |
|  | Baseline characteristics | | |  |  |  |
|  | Sex, male, N (%) | | | 42 (64.6) | 52 (54.7) | 0.253 |
|  | Median age (years) at HM OFC (IQR) | | | 3.4 (2.1–5.4) | 2.3 (1.6–5.3) | 0.161 |
|  | Atopic dermatitis, N (%)^†^ | | | 3 (4.6) /16 (24.6) / 46 (70.8) | 12 (12.6) / 43 (45.3) / 40 (42.1) | **0.002*** |
|  | Asthma, N (%)^†^ | | | 42 (64.6) / 4 (6.2) / 19 (29.2) | 81 (85.3) / 4 (4.2) / 10 (10.5) | **0.005*** |
|  | Pollen allergy, N (%)^†^ | | | 31 (47.7) / 2 (3.1) / 32 (49.2) | 60 (63.2) / 2 (2.1) / 33 (34.7) | 0.118 |
|  | Animal dust allergy, N (%)^†^ | | | 38 (58.5) / 4 (6.2) / 23 (35.4) | 70 (73.7) / 3 (3.2) / 22 (23.2) | 0.124 |
|  | Other food allergies, median (IQR) | | | 2.0 (1.0–2.0) | 1.0 (0.0–2.0) | **0.027*** |
|  | | Atopic dermatitis in family, N (%) | | 44 (67.7) | 60 (63.2) | 0.614 |
|  | | Asthma in family, N (%) | | 19 (29.2) | 30 (31.9) | 0.861 |
|  | | Allergies in family, N (%) | | 49 (75.4) | 73 (76.8) | 1.00 |
|  | | Tobacco smoking in family, N (%) | | 3 (4.6) | 9 (9.5) | 0.363 |
|  | Median HM OFC cumulative dose, mg | | |  |  |  |
|  | ≤ 3-year-old (IQR) | | | 1935 (566–1935) | 1935 (1935–3175) | **0.032*** |
|  | >3-year-old (IQR) | | | 1935 (385–5035) | 5035 (2000–5035) | **0.003*** |
| FM OFC positive, N (%) | | | | 64 (98.5) | 81 (87.1) | **0.015** |
| HM OFC positive, N (%) | | | | 36 (64.3) | 14 (19.7) | **<0.001*** |

eTable S3. Baseline characteristics, median values of SPTs and sIgEs, age-related HM cumulative doses, and OFC outcomes according to BAT status (A) and avidity status (B). BAT status was determined either as successful or not successful (including non-responsive, hyperreactive, or not performed). A BAT result was considered non-responsive if activation rate of basophils was <10% with the positive control FcƐRI monoclonal antibody and hyperreactive if spontaneous activation at baseline without stimulation exceeded 5%. Avidity measurements were considered successful or not successful (unreliable or not performed). Unreliable avidity results had baseline absorbances <0.2.

Other food allergies screened for: egg, any nut, fish, wheat, soy.

Missing data (A): asthma and allergies in family for 1 child in the successful BAT group; SPT measurements from 1 child in the successful and 3 children in the group with no successful BAT measurements.

Missing data (B): SPT measurements from 2 children both in the successful group and the group without successful avidity measurements. Asthma in family for 1 child in the group with no successful avidity measurements and allergies in family for 1 child in the successful avidity measurements group.

sIgE: specific immunoglobulin E, kU/L: kilounits per liter, IQR: interquartile range; BSA, bovine serum albumin, mm: millimeter, HM OFC: heated milk oral food challenge, FM OFC: fresh milk oral food challenge

* Denotes a statistically significant (p < 0.05) difference (without Bonferroni correction).

^†^ Never/previously/currently
