# Supplementary material for: Casein sIgE avidity and milk basophil reactivity in children with cow's milk allergy
Source: Pediatr Allergy Immunol. 2026 Aug 18;37(8):e70464. doi: 10.1111/pai.70464 (PMC13486920; doi:10.1111/pai.70464)
Supplement: Supplementary file 5 [file PAI-37-e70464-s006.docx]

A)	B)

|  | | FM OFC+ (BAT, N=51) | FM OFC + (avidity, N=64) |
| --- | --- | --- | --- |
| Reaction severity | |  |  |
|  | Mild | 41 (80.4) | 48 (75.0) |
|  | Moderate | 5 (9.8) | 8 (12.5) |
|  | Severe | 5 (9.8) | 8 (12.5) |
|  | Anaphylaxis | 2 (3.9) | 4 (6.3) |
| Symptoms | |  |  |
|  | Skin | 41 (80.4) | 47 (73.4) |
|  | Upper respiratory | 10 (19.6) | 23 (35.9) |
|  | Lower respiratory | 12 (23.5) | 14 (21.9) |
|  | Subjective gastrointestinal | 16 (31.4) | 20 (31.3) |
|  | Objective gastrointestinal | 7 (13.7) | 12 (18.8) |
|  | Tingling of mouth | 19 (37.3) | 33 (51.6) |

|  | | HM OFC+ (BAT, N=21) | HM OFC + (avidity, N=36) |
| --- | --- | --- | --- |
| Reaction severity | |  |  |
|  | Mild | 14 (66.7) | 22 (61.1) |
|  | Moderate | 5 (23.8) | 7 (19.4) |
|  | Severe | 2 (9.5) | 7 (19.4) |
|  | Anaphylaxis | 2 (9.5) | 4 (11.1) |
| Symptoms | |  |  |
|  | Skin | 16 (76.2) | 19 (52.8) |
|  | Upper respiratory | 7 (33.3) | 15 (41.7) |
|  | Lower respiratory | 6 (28.6) | 11 (30.6) |
|  | Subjective gastrointestinal | 5 (23.8) | 6 (16.7) |
|  | Objective gastrointestinal | 2 (9.5) | 2 (5.6) |
|  | Tingling of mouth | 11 (52.4) | 16 (44.4) |

eTable S2. The severity of reactions and the rate of different symptoms experienced during positive FM OFCs (A) and positive HM OFCs (B) in children with reliable BAT results and reliable avidity measurements. Severity assessment is according to the modified Sampson’s severity score,^1^ and anaphylaxis is defined according to Sampson et al.^2^

1.	Nieminen O, Palosuo K, Kukkonen K, Mäkelä M. Comparison of Severity Scorings in Oral Food Challenges With Cow’s Milk. J Investig Allergol Clin Immunol. 2024;34(1):71–3.

2.	Sampson HA, Muñoz-Furlong A, Campbell RL, Adkinson NF, Bock SA, Branum A, et al. Second symposium on the definition and management of anaphylaxis: Summary report—Second National Institute of Allergy and Infectious Disease/Food Allergy and Anaphylaxis Network symposium. J Allergy Clin Immunol. 2006 Feb 1;117(2):391–7.
